# Reassessing the terminal ballistic performance of trilobate and quadrilobate arrow points on Iron Age battlefields

**DOI:** 10.1371/journal.pone.0288483

**Published:** 2023-07-26

**Authors:** Devin B. Pettigrew, William Taylor

**Affiliations:** Department of Anthropology, University of Colorado Boulder, Boulder, CO, United States of America; University of California Santa Cruz, UNITED STATES

## Abstract

In the Eurasian Iron Age arrow points comprise a prominent class of artifact. Projectile experiments are useful for studying the ballistic performance of ancient arrow points and implications of arrow point innovations in warfare and shifting socio politics in Eurasia. However, when projectile experiments are not representative of past weapon use, they can lead to misinterpretations of the archaeological record. Notable problems arise when homogeneous target simulants used in controlled experiments are not representative of the targets past weapons were designed to encounter. This article explores the relationship between arrow point morphology and design choices in the Iron Age using different target media. Shooting arrow points into pottery clay leads to the conclusion that more blades reduced penetrating performance on ancient battlefields, but a very different result obtains by shooting the same points into thick tooling leather as a simulant for leather body armor. The results help explain patterns observed in the Eurasian archaeological record, where trilobate arrow points–initially developed by lightly armored horse archers on the Eurasian steppe–were increasingly adopted by a wide range of societies across Eurasia throughout the Iron Age.

## 1. Introduction

**Fig 1:**
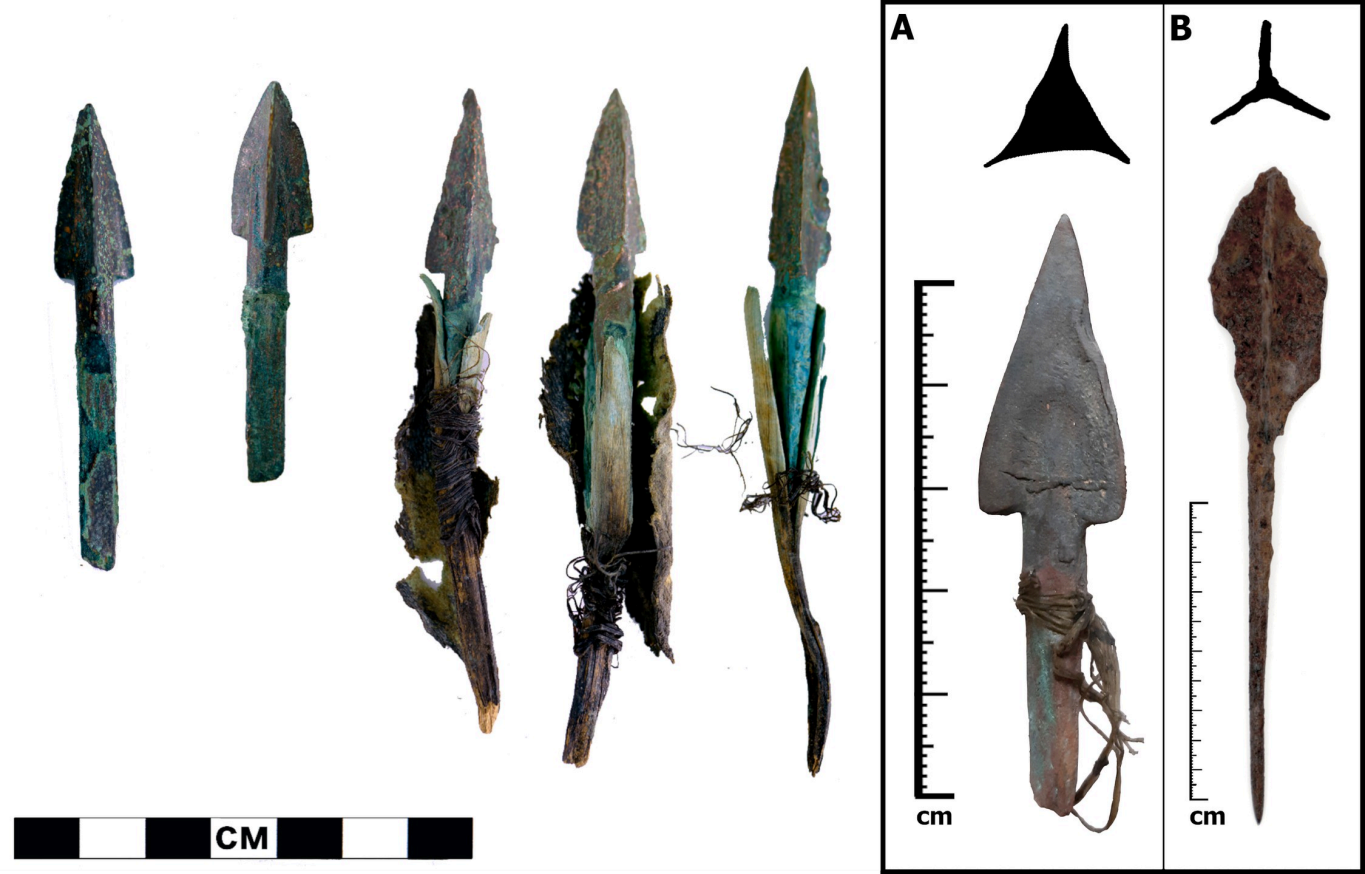
Tanged triangular and trilobate arrow points from northern Mongolia: (left) bronze points from the early mid-first millennium BCE site of Jargalantyn Am, photo by J. Bayarsaikhan; (right, A) early first millennium BCE bronze and (right, B) first millennium CE iron points from Tsengel Khairkhan, photos by N. Jarman and P. Bittner respectively [after 6].

**Fig 2:**
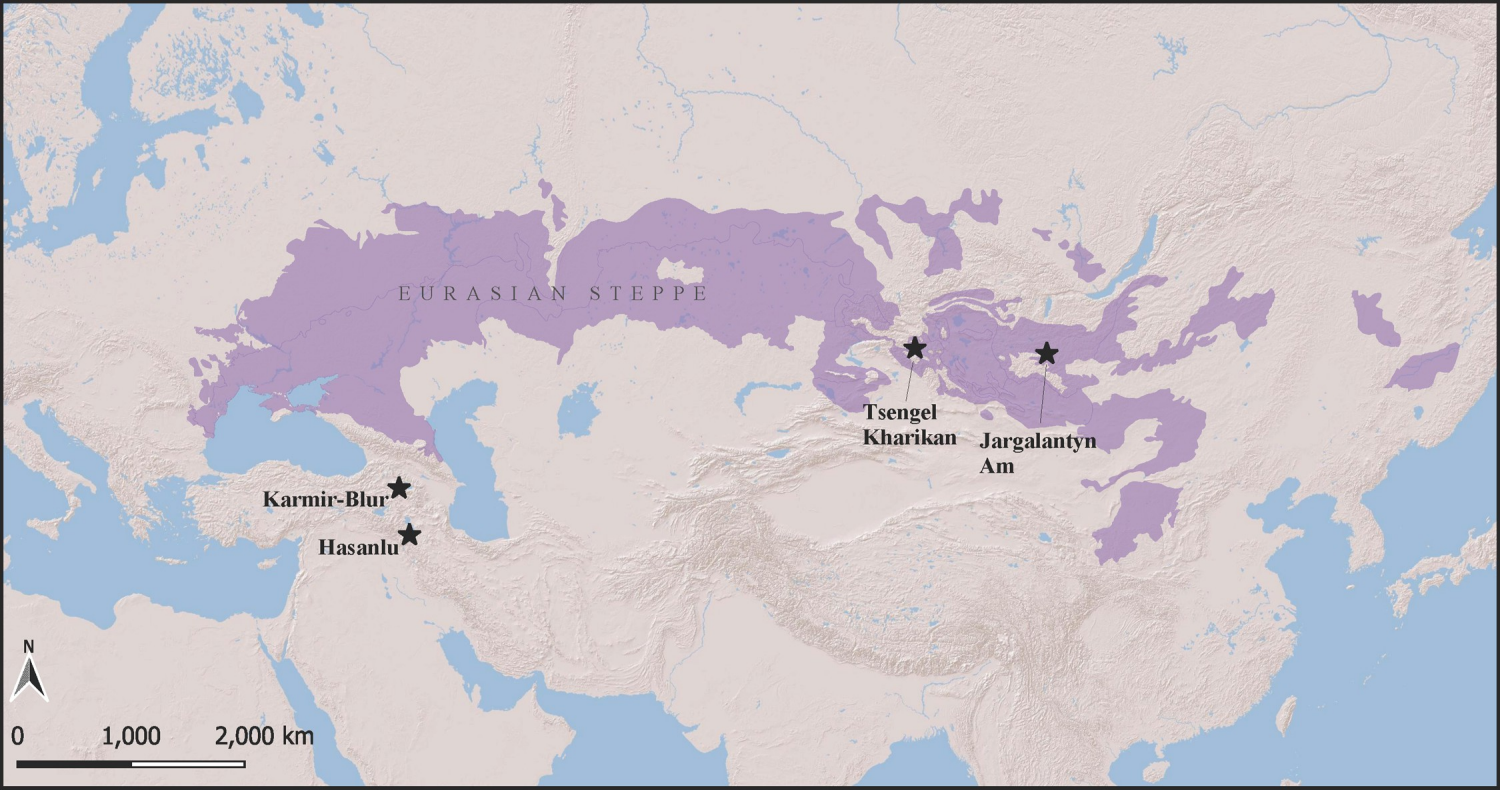
Locations of sites and geographic regions mentioned in the text. Produced in QGIS 3.3 (https://www.qgis.org/). Steppe spatial data retrieved from https://www.worldwildlife.org/publications/terrestrial-ecoregions-of-the-world.

On the steppes of Eurasia during the early 1^st^ millennium BCE, arrow points were a primary tool of warfare during a shifting period of technology and geopolitics. Current evidence suggests that trilobate (three-blade) metal arrow points (Fig 1) first appeared on the Eurasian steppe (Fig 2) by the early 1^st^ millennium BCE [1–3], after which they are found in both burials [4, 5] and hunting contexts [6] from Mongolia to the Black Sea. By the mid-7^th^ century BCE, cast bronze socketed bilobate (two-blade) and trilobate arrow points spread southward into the Near East, where they were used in attacks against fortified towns and were incorporated into local weapon arsenals alongside earlier tanged iron bilobate arrow points [3, 7, 8]. Following this introduction, quadrilobate (four-blade) bronze points were also manufactured in limited quantities in the Near East [3, 7] and some quadrilobate iron points are suggested from corroded finds [7, 9]. Trilobate arrow points then spread rapidly throughout the Near East and beyond, appearing in Hellenic contexts, among others [2, 3, 10, 11]. By the 6^th^ century BCE, socketed trilobate points appeared in the Levant, where they were most likely introduced by Mesopotamian intermediaries [12], as well as in the Mediterranean and Central Europe [9, 11]. Despite the significantly greater challenge of hand-forging trilobate blades from iron relative to bilobate forms [3, 13], tanged iron trilobate points appeared in the Near East by the Achaemenid period [1] and spread across Eurasia during the Iron Age [1, 2, 9, 14, 15]. In the Roman military, iron trilobate points–used initially by foreign units of archers from the east–came to comprise the most common arrow point type after the 3^rd^ century AD among Roman military archers across the empire [2, 9, 16].

Many scholars have used the presence of these arrow point types as indicators of foreign attacks or settlement by foreign ethnic groups, such as the Scythians and Cimmerians [see 3, 7, 8, 17, 18]. However, Derin and Muscarella [8] review how using socketed bronze points as ethnic identifiers in the destruction of the kingdom of Urartu has led to a chaotic series of interpretations, since even in the context of a destroyed fort it is often impossible to tell whether an arrow was shot by an attacker or defender. A similar situation is described by Dušek [17], who challenges the attribution of socketed bronze points to the presence of Scythians in Central Europe on the basis of the extremely wide distribution of these points and lack of sufficient contexts of finds. Szudy [3] reviews evidence suggesting that steppe arrow points may have been adopted quickly in the Near East in the early 1st millennium BCE. Forged iron skeuomorphs of northern socketed bronze points were discovered at Hasanlu IVB from the 9^th^ century BCE, where they were found together in fused clusters–likely representing the remains of quivers stockpiled by defenders–that also contained various types of tanged iron and bronze points [19]. Along with other early Scythian-type artifacts, bronze bilobate and trilobate points were also discovered together with iron bilobate points in the magazines at Karmir-Blur, indicating fairly intense cultural interactions with the steppes [20]. The spread of these points may have been facilitated in part by trade and political alliances between steppe and Near Eastern cultures [7].

Significantly, some scholars have characterized changes in arrow point designs as part of an *arms race* in the ancient Near East, driving the development of body armor as well as more effective weapons to combat armored opponents [21–23]. As depicted in artwork and recorded in surviving ledgers, combatants on ancient Bronze and Iron Age battlefields frequently wore armor, sometimes including or even comprised entirely of metal scales sewn to a backing, but the majority of armor worn by common soldiers was constructed of more affordable fabric or leather [21, 24–27]. Due to the highly perishable nature of such armor, intact examples are rare, making it challenging to trace its age or distribution [26, 28, 29]. A variety of leather armor most likely existed [21], with the simplest forms comprised of single-piece layers, such as armor worn historically for arrow defense in Siberia [29], and more complex forms of overlapping rawhide or leather scales sewn to a leather backing [21, 26]. Relative to metal scales, leather scale armor was not only cheaper and easier to produce, but it also had the benefit of being lighter–a particularly valuable consideration for archery combat on horseback, which became an important form of military combat across Eurasia during the first millennium BCE [30]. In the early 1^st^ millennium BCE, new techniques for mass-producing leather scale armor–associated with the Neo-Assyrian empire–spread as far away as Xinjiang in northwest China [26]. Artwork of the Neo-Assyrian period supports this idea, depicting entire armies clad in armor [24, 25]. Across much of Inner Asia, light armor and clothing made of skin and furs, along with lamellar armor made of organic plates, tended to be favored in mounted combat from the first millennium BCE [31, 32]. These types of *soft armor* persisted to a late date–leather and dense fabric armors, in some cases as composite forms or worn as supplements to metal armor, continued to be worn in Europe well into the medieval period [33, 34].

If arrow points were initially developed on the steppes specifically for their improved ability to defeat soft armor and penetrate the body of a combatant, this carries significant techno-functional implications that could influence their adoption outside the steppes [35], providing additional evidence to challenge simplistic ethnic attributions to arrow point types in archaeological assemblages. However, experiments to test the performance of ancient weapons are commonly problematized by inappropriate targets [36]. In a recent controlled experiment at Kent State University, Mullen and colleagues [37] shot replica bilobate and trilobate cast bronze points into pottery clay as a simulant for *flesh* (i.e., muscle tissue, mixed with fat and other soft tissue [see also, 38]). The results showed that trilobate points penetrated more shallowly than bilobate points in clay, leading to questions about why trilobate arrow points ever became popular in the ancient world [37]. However, a further experiment to validate pottery clay as a flesh simulant demonstrated that clay does not capture the same features of arrow point efficacy as soft biological tissues [36]. Although Pettigrew and Bamforth [36] found that dull points decelerated more rapidly penetrating leather, sharp and dull broadheads of the same shape and mass penetrated the same depth in clay. Furthermore, a conical target point, as well as a completely blunt point specifically designed not to penetrate skin but to deliver a shock to small game, both penetrated significantly deeper than a sharp two-blade steel broadhead into clay [36]. The surprising effectiveness of the blunt point reproduced the same effect observed by Ankersen et al. [39] for blunt and sharp steel knives pressed into Roma Plastilina modeling clay. This confirms the markedly different density, flow behavior [40, 41], and friction versus fracture toughness [42, 43] of clay relative to soft biological tissues or leather. These results are at odds with a previous test of medieval arrow points shot into reconstructed leather and composite linen armor, where edge sharpness was identified as the most crucial factor for penetration, and wider blades were found to reduce friction on the trailing shaft by cutting a larger hole in the target [33]. In further disparity with clay, a conical target point failed to penetrate these targets [33].

Importantly, terminal ballisticians also recognize that large masses of flesh, which artificial targets are generally intended to simulate, are not the usual targets of projectile attacks. Rather, projectiles are typically aimed at the torsos of prey animals protected by hide [44, 45], or people wearing clothing or armor [42, 46, 47]. Projectiles in these contexts must first penetrate an outer layer of tougher material before they can damage softer and less resistive tissues underneath. Results of controlled experiments against homogenous flesh simulants (typically 10% ordinance gelatin) are also difficult to extrapolate to heterogenous biological structures: skin, subcutaneous flesh interconnected with skeletal bone, and less-resistive internal organs [40, 41, 46, 48, 49]. Understanding how target simulants compare with real targets, therefore, requires careful comparison with real wounds and experiments in more realistic targets [40], and when studying the effects of weapons against body armor or clothing, simulating the specific properties of those materials is of seminal importance to understanding weapon efficacy [33, 47, 50–53].

Understanding the stylistic or utilitarian attributes of arrow points can contribute to the discussion of their spread and possible representations of identity in the archaeological record [e.g., 54], but only when interpretations are drawn from experimental results in scalable targets [36]. To reassess the performance of multiblade arrow points on ancient battlefields, then, we present data from controlled experiments in which we repeated the test of shooting multiblade arrow points into pottery clay [37] and contrast the results with shots through a thick tooling leather as a generalized leather body armor simulant [sensu 21, 33]. In contrast to naturalistic experiments using reconstructed bows and arrows shot in conditions that more closely mimic the highly variable nature of real use [e.g., 55, 56], the approach we use here carefully controls for variables such as impact velocity, the weight of the arrow shaft, the specific shape and surface treatment of the arrow point, and importantly, the sharpness of the tip and cutting edge. While both naturalistic and controlled approaches are useful for interpreting the archaeological record [57–61], this approach allows us to isolate the effect of adding more blades to the penetrating performance of arrow points in clay and leather. Three fundamental variables of arrow terminal ballistics (the ballistics of target impact and penetration) are considered: (1) penetration depth, (2) force of target resistance (F_r_) to cutting, and (3) force of drag (F_d_) on the trailing shaft.

## 2. Methods

### 2.1 Arsenal

**Fig 3:**
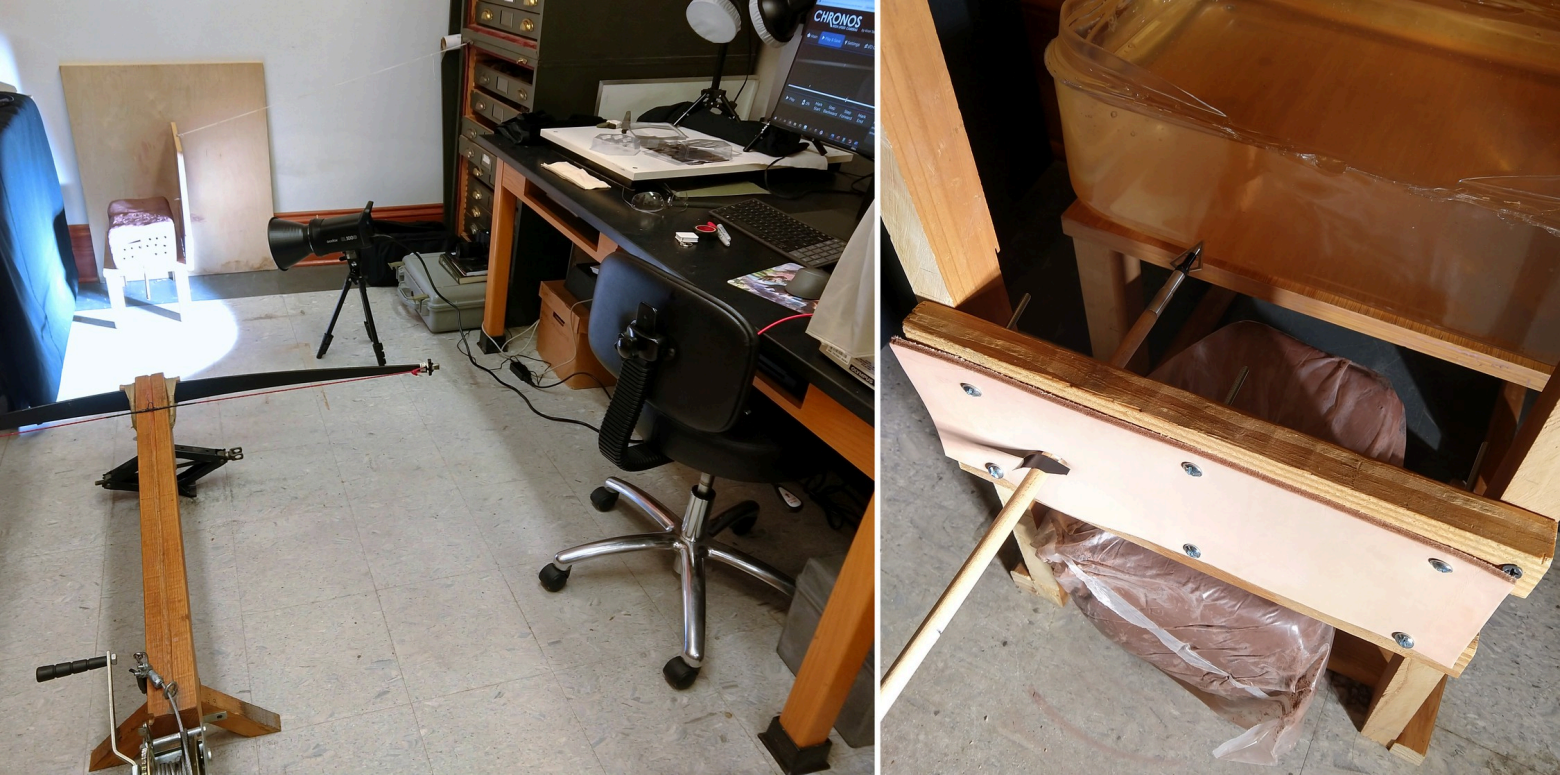
The design of the experiment: (left) shooting a clay target, and (right) a two-blade broadhead penetrating one layer of tooling leather, which has stopped prior to hitting a gel backing.

**Fig 4:**
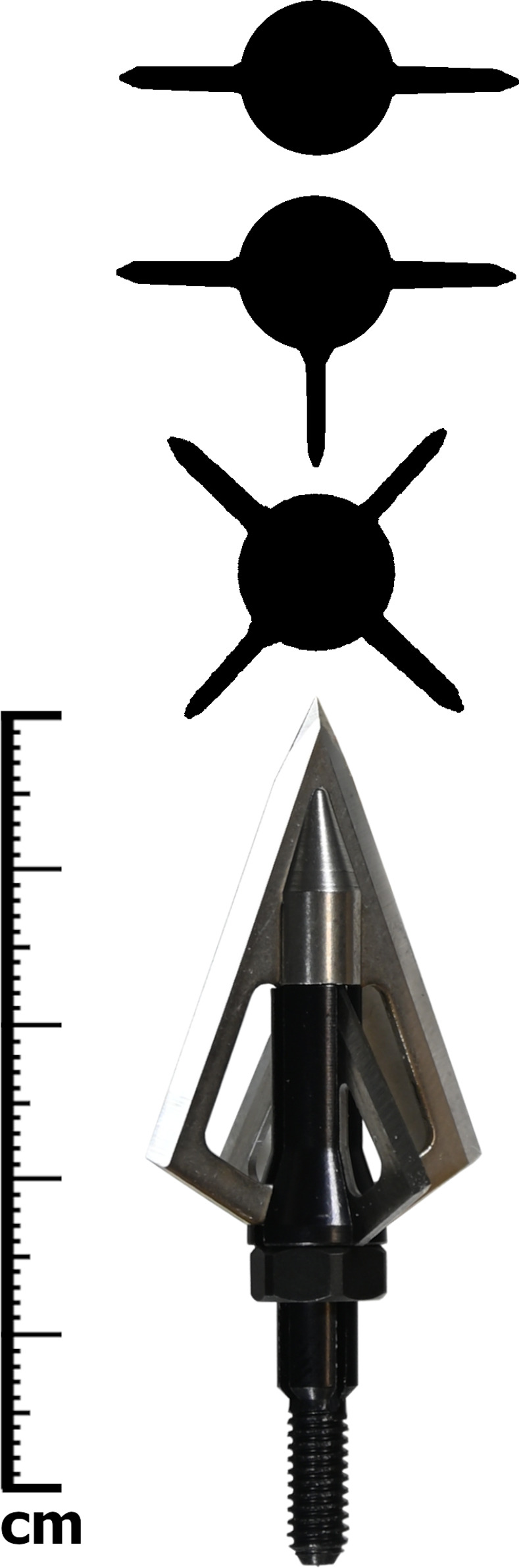
A Wasp Sharpshooter broadhead with removable blades and three tested blade configurations shown in cross-sectional profile.

The experiment deployed a crossbow comprised of a steel prod lashed to an oak stock and drawn with an archery trigger attached to a hand-crank winch (Fig 3). Two identical bolts were constructed of 9.5 mm diameter poplar (Populus sp.) dowels and 3-Rivers aluminum adapters to hold screw-in arrow points. Excluding points, the bolts measured 618 mm long and weighed 24 g. Three Wasp Sharpshooter broadheads numbered BH7-9 as part of a larger test arsenal [62] and weighing 6.5 g each were chosen as test points (Fig 4). Two smaller blades weighing 0.4 g each could be removed from the sides of the broadheads, allowing them to be shot in two, three, and four blade configurations. This produced a non-radial orientation in the three-blade configuration, but allowed us to carefully control potential impacts of differential edge sharpness (section 2.3). The broadheads came sharp from the factory and were not resharpened over the course of the experiments.

### 2.2 Targets

We chose a stiff 3.2–3.6 mm thick vegetable-tanned cowhide tooling leather as a generalized leather armor simulant [sensu 21, 33], which we found to be highly resistive to cutting even with a sharp knife. Strips 100x305 mm wide were attached to a rigid wooden stand. After initially cutting through leather, the bolts could traverse 300 mm of open space before impacting a block of synthetic ballistics gelatin (Perma-Gel), which merely served to catch the bolt (Fig 3). We chose this target configuration–testing the leather without a flesh simulant backing–to avoid problems with the non-scalability of gel or clay as flesh simulants for arrows even when overlain by a leather exterior [36].

Additionally, 11 kg blocks of moist pottery clay were purchased from Rocky Mountain Clay in Denver, Colorado. Although specific formulas are not provided for commercial reasons (Lynn Williams, personal communication, 2021), a low-fire clay without grog (Red Rock Red Smooth) was chosen to reproduce as closely as possible the clay used in the previous experiments. Two clay blocks were pressed together to form a homogenous target and reformed by throwing several times on a countertop after a round of shooting. The blocks were kept in plastic bags when not in use to reduce moisture loss.

### 2.3 Shooting

We positioned the crossbow 950 mm from the target face to allow room for the bolt to clear the stock. A consistent draw length of 550 mm produced a moderate arrow velocity (mean = 35.2 m/s, std dev = 1.1 m/s, n = 67; [63]). To even out any effects from differences in edge or tip sharpness between the broadheads, we shot them sequentially in two, three, and four blade configurations through leather, so that each blade configuration was equally represented by each broadhead.

First, the broadheads were shot three times in each configuration sequentially through multiple layers of leather for measuring cutting resistance and penetration depth. Initially, three layers of leather were bolted to the stand, but the first shot with BH7 only penetrated to the base of the blades. BH8-9 were then shot three times each and BH7 twice more through two layers of leather (the second shot with BH8 partially cut through a previous shot and was discarded). The small sample size for this test was chosen to reduce effects of edge attrition for the next component of the experiment, shooting the broadheads ten times in each configuration sequentially through one layer of leather for measuring drag on the trailing shaft. Finally, each broadhead was set in either a two (BH7), three (BH8), or four blade (BH9) configuration and shot sequentially ten times each into clay. We removed clay from the broadheads and bolt with fingertips and a damp sponge between shots.

### 2.4 Data collection and analysis

**Fig 5:**
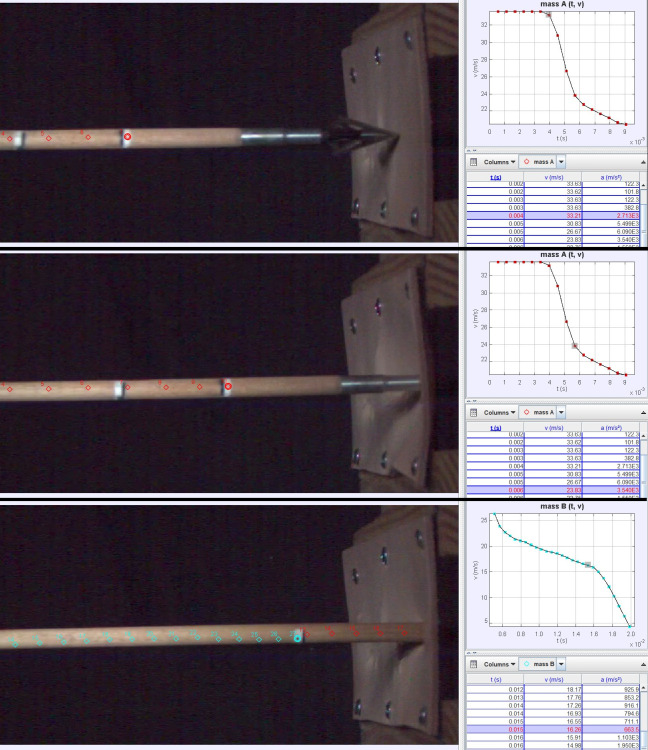
Screen captures showing Tracker analysis of a single shot through one layer of 3 mm tooling leather. Top and middle screens show the measurement of V_i_ and V_f_ for calculating F_r_ on the broadhead from point mass A, while the bottom screen shows the last frame in a sequence of *n* = 15 deceleration readings averaged to calculate F_d_ on the wooden shaft from point mass B.

To measure penetration, we placed a piece of electrical tape on the bolt at the exterior of the target, extracted the bolt, and measured from the broadhead tip to the edge of the tape to the nearest mm with a ruler. To record velocity and deceleration through leather, a Kron Technologies Chronos 1.4 high speed camera connected to a nearby computer filmed video in 640x240 pixel resolution at 8810.57 frames/second orthogonal to the shooting line. Orthogonality was checked by placing a 10 mm grid on a box perpendicular to the target face and viewing it through the camera [sensu 64]. Three marking locations made on the bolt with white reflective paint and red and black marker provided points to track and scales to calibrate the video. We performed video analysis in the open-source Tracker program (https://physlets.org/tracker/): First, the moment the base of the broadhead disappeared into the target was chosen as the end of an increased step sequence of five video frames (Fig 5). Point masses (A and B) were then created and markers were carefully centered over small red dots on the bolt. Initial velocity (V_i_) was averaged from multiple Tracker readings prior to target impact, while final velocity (V_f_) comprised the reading immediately after the full length of the broadhead up to the base had entered the target (PEN_length_). Penetration duration (PEN_time_ = PEN_length_/V_i_), deceleration (a = (V_i_-V_f_)/PEN_time_), and force of target resistance (F_r_ = a*m) were calculated in a Microsoft Access database. To calculate force of drag (F_d_) on the trailing shaft, several deceleration values calculated by Tracker using a finite differences algorithm over four marked points [65] were averaged.

In the following, we present mean comparison tests for the three broadhead configurations using a two-tailed significance level of 0.05. We compare penetration for *n* = 30 shots into clay, and F_r_ and F_d_ for *n* = 30 shots into one layer of 3 mm leather. We performed the statistical analysis in JMP [66]. Effects are considered significant at the p≤0.05 level.

## 3. Results

### 3.1 Kinetic energy and momentum differences

Removal of the 0.4 g blades resulted in subtle differences in mass of the complete bolt for the three broadhead configurations. It is therefore warranted to test for impacts of two variables on projectile penetration: Kinetic energy (KE = 0.5m*v^2^) captures the ability of the projectile to create new surface areas within a solid target by fracturing through it, and momentum (P = m*v) captures the ability of the projectile to resist changes in its state of motion as a result of forces acting against it [43, 62, 64]. One-way ANOVA tests show that differences in kinetic energy (f(2, 64) = 0.5314, p = 0.5904) and momentum (f(2, 64) = 2.8898, p = 0.0629) are not statistically significant between the blade configurations. Moreover, mean kinetic energy and momentum are only subtly different between the configurations (Table 1).

**Table 1:** Kinetic energy (KE) and momentum (P) differences between broadhead configurations.

		**KE (J)**	**P (kg-m/s)**
**2 blade**	mean	18.5	1.05
	std dev	1.2	0.03
	min	16.9	1.00
	max	20.0	1.09
	*n*	23	23
**3 blade**	mean	18.8	1.06
	std dev	1.2	0.03
	min	16.8	1.01
	max	20.2	1.10
	*n*	22	22
**4 blade**	mean	18.9	1.07
	std dev	1.2	0.03
	min	17.2	1.02
	max	20.6	1.12
	*n*	22	22

### 3.2 In pottery clay

**Fig 6:**
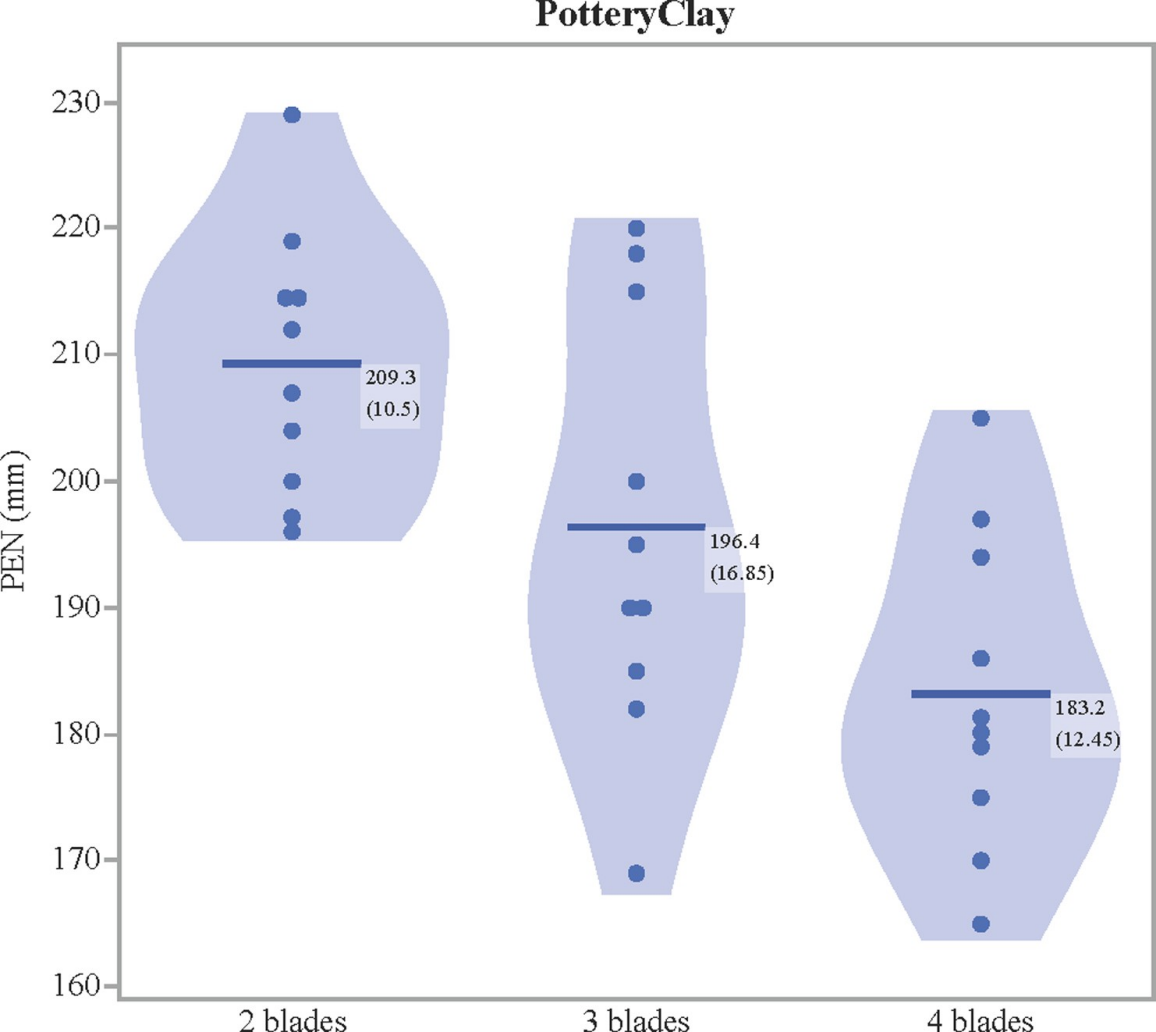
Penetration depth in pottery clay. Means followed by standard deviations accompany each group.

Results of penetration depths between the three blade configurations are presented in Table 2. Corroborating the previous experiment [37], the use of more blades resulted in a slight reduction of penetration in these trials: relative to two blades, three blades reduced penetration by 6.2% and four blades by 12.5% into clay (Fig 6). A one-way ANOVA reveals a statistically significant difference between the penetration means (F(2, 27) = 9.3045, p = 0.0008).

**Table 2:** Penetration depth (PEN, mm) and forces of target resistance (F_r_, N) and drag (F_d_, N) in all targets.

		**Clay**	**3mm Tooling**			**3mm Toolingx2**	
		**PEN**	**F**_**r**_	**F**_**d**_	**PEN**	**F**_**r**_	**PEN**
**2 blade**	mean	**209**	**235**	**48**	**263**	**623**	**66**
	std dev	10.5	49.2	12.2	46.3	28.9	2.5
	min	196	171	32	188	590	63
	max	229	314	68	300	643	68
	*n*	10	10	10	10	3	3
**3 blade**	mean	**196**	**217**	**27**	**300**	**671**	**75**
	std dev	16.8	23.7	6.2	0.0	87.7	9.9
	min	169	177	21	300	609	68
	max	220	254	39	300	733	82
	*n*	10	10	10	10	2	2
**4 blade**	mean	**183**	**235**	**21**	**300**	**732**	**82**
	std dev	12.5	32.0	4.5	0.0	5.8	4.2
	min	165	193	15	300	728	79
	max	205	294	29	300	737	85
	*n*	10	10	10	10	2	2

### 3.3 In 3 mm tooling leather

**Fig 7:**
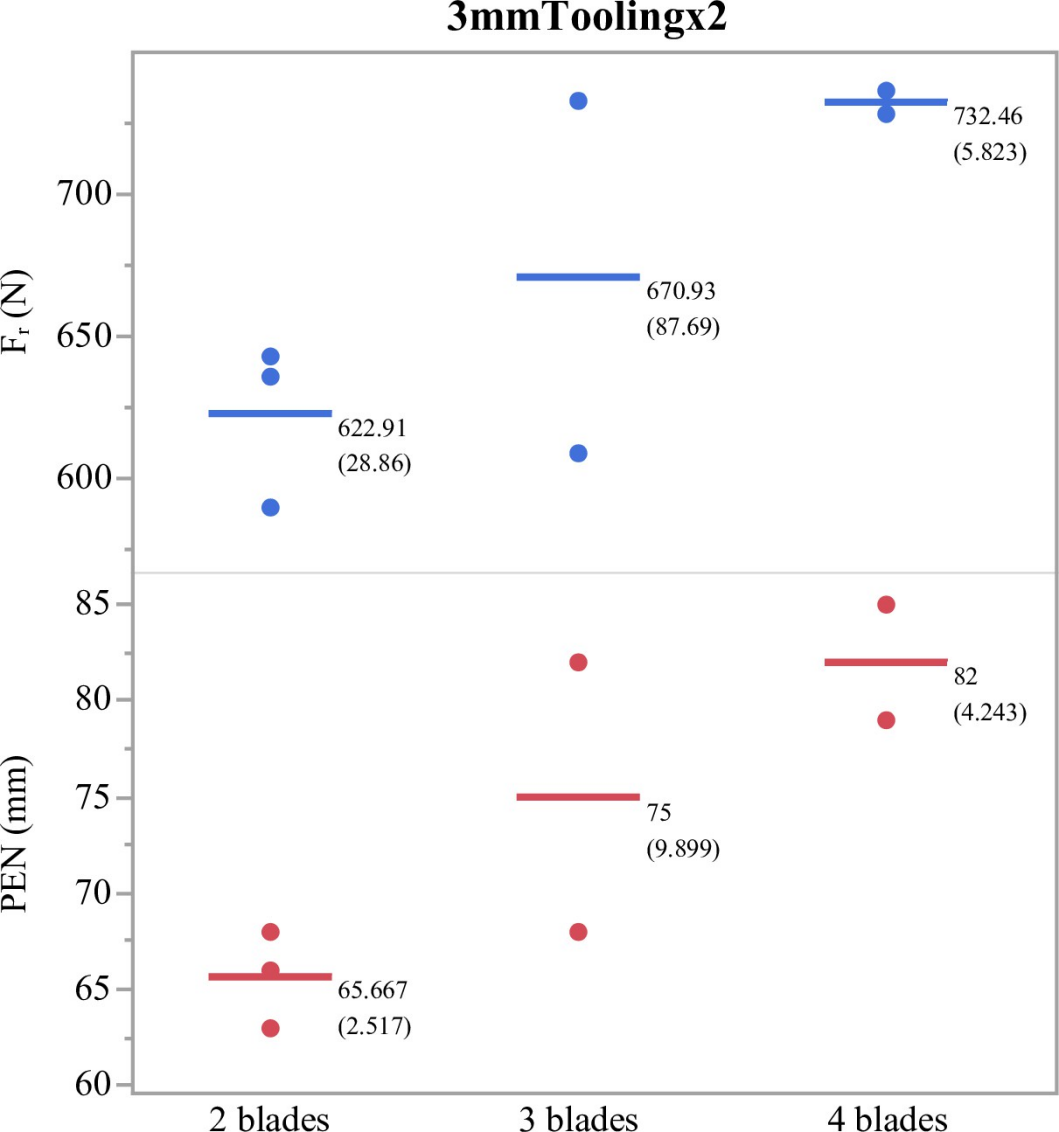
Force of resistance and penetration depth through two layers of 3mm tooling leather. Means followed by standard deviations accompany each group.

The results of shooting through two layers of stiff leather show that, relative to two blades, three blades increased mean force of target resistance (F_r_) by 7.7% and four blades by 17.6%. However, in contrast to clay, three blades increased mean penetration depth by 14% and four blades by 25% (Fig 7 and Table 2).

**Fig 8:**
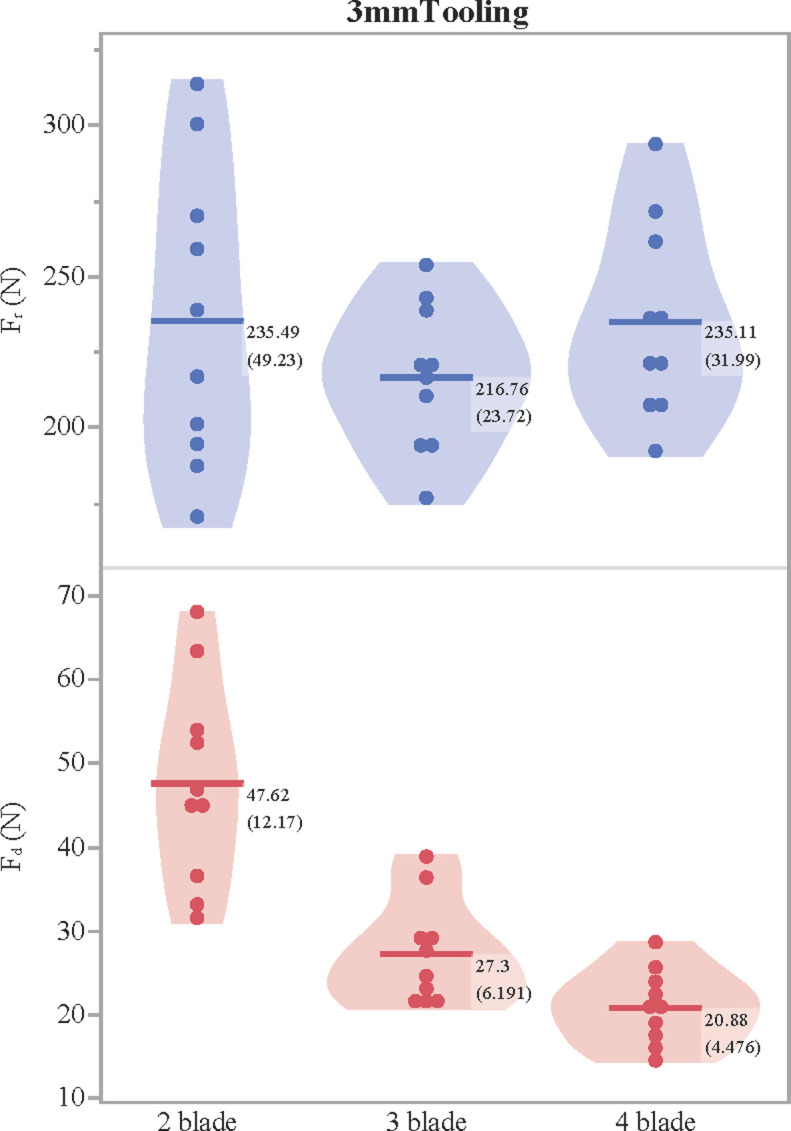
Force of resistance (F_r_) and drag on the trailing shaft (F_d_) through one layer of 3 mm tooling leather. Means followed by standard deviations accompany each group.

Next, a single layer of leather was bolted to the stand. Here, one-way ANOVA tests do not reveal a statistically significant difference in F_r_ between the three broadhead configurations (f(2,27) = 0.8564, p = 0.4359). F_r_ is unexpectedly high and most variable for the two-blade configuration (Fig 8), but when only shots with three and four blades are compared, a two sample t-test still does not find a statistically significant difference between broadhead configuration and Fr (t[16.5] = 1.4570, p = 0.1638). However, a one-way ANOVA does find that differences in force of drag (Fd) on the trailing shaft are statistically significantly different for all three configurations (f(2, 27) = 28.3013, p<0.0001): relative to two blades, mean Fd was reduced by 43% with three blades and by 56% with four blades (Fig 8 and Table 2). Furthermore, while broadheads with three and four blades always traversed the 300 mm distance and penetrated up to their bases into the gelatin after passing through one layer of leather, in five of the ten shots, broadheads in the two-blade configuration stopped after passing only 188–270 mm through the leather, prior to hitting the gelatin (Fig 3). This helps corroborate the penetration results from the small sample of shots in two layers of leather. Note, however, that F_r_ means are more than doubled in two layers relative to one layer of leather, indicating that the relationship between resistance and the number of layers is not linear.

Since in the three-blade configuration the blades were not evenly spaced (i.e., radial; Fig 4), the bolt was observed to flex and slightly deflect ca. 2 mm (measured with the measuring tool in Tracker) from its initial trajectory into the wider area of the cut. This may have resulted in slightly greater forces during initial penetration of leather, relative to three radial blades.

## 4. Discussion

Results of these experiments demonstrate that three and four-blade arrow points, while less effective at penetrating clay, are significantly better at penetrating thick leather than two-blade arrow points. The reasons for this can be understood through the principles that govern arrow penetration: the kinetic energy and momentum of the arrow shaft, the cutting/piercing efficacy of the armature, drag occurring at the armature haft and trailing shaft, and the specific material properties of the target [43, 44, 62, 64, 67, 68]. Although our experiments controlled for kinetic energy and momentum, the other variables (penetrating efficacy, drag, and properties of the target) are highly relevant to understanding our study results.

Although larger cutting armatures can increase the force of cutting, they can also reduce friction on trailing hafts and shafts by opening a larger hole in the target [33, 44, 68, 69], and if arrows with more blades can penetrate effectively, they result in larger wounds, increasing the deadliness of an arrow and the difficulty of treating a wounded combatant [5, 9, 44, 70]. These effects apply to targets like leather or skin, but are not captured in experimental conditions using clay, which tends to capture friction on larger surface areas [71], even including the blade flanks of sharper tools [39]. For this reason, optimization for penetration in clay targets is biased towards smaller armatures and even blunt armatures that push material aside and reduce friction on the trailing shaft [36, 39]. The ability of sharp tips and edges to efficiently penetrate a target with high fracture-toughness [43] and to cut a path through a target is not captured in clay, although sharpness is clearly important to penetration through thick leather, bronze scales, composite linen [21, 27, 33, 36], modern soft body armor, clothing, and biological tissues [42, 72–74].

Although the use of modern broadheads and stiff tooling leather in this experiment did not directly replicate the functional performance of cast bronze or forged iron arrow points, nor did our experiment capture the extreme range of arrow ballistics or target materials that occurred on ancient battlefields, using these points and targets nonetheless introduced a greater degree of reproducibility and control, allowing points of the same shape and mass to be easily interchanged on the same shaft and with extra blades that could be removed. This allowed us to isolate the effect of adding extra blades while balancing unwanted effects of differential edge sharpness and edge attrition over the course of the experiment. This is important, as sharpness, which is a problem of the microscopic radius of the edge, is challenging to measure or consistently reproduce [see 43, 75], yet as we have noted, sharpness is an essential factor in an arrow’s ability to penetrate armor and into the body of a combatant. In the three-blade configuration the orientation of the blades was also not radial, however, what negative impact this had is not captured in the results, where three and four blades decreased penetration into clay as expected given their greater surface area [37, 39, 76], but increased penetration through leather by decreasing drag on the trailing shaft [33]. The reduction in drag from a larger hole cut in leather should also not change whether an armature was made of bronze, iron, or steel, barring shape or size differences, although an armature’s ability to initially defeat armor of varying hardness and fracture toughness could change. These results further demonstrate that the efficacy of arrow point design is dependent on the specific properties of the targets arrows most frequently encounter.

It is apparent from surviving artwork, ledgers, and archaeological finds that archers in the ancient world regularly targeted opponents wearing body armor, with leather likely being the most common armor material in the ancient Near East [21, 26]. Leather and fabric armor was also popular in Europe into the medieval period [33, 34]. The ability of a point to efficiently cut a hole and reduce drag on the trailing shaft [44] is clearly important in this context. Hulit found that dull bronze arrow points shot with a replica Assyrian composite bow would not penetrate thick leather or replica bronze scales [21]. Similarly, Jones found tip and edge sharpness to be the most important factor for arrow penetration through dense linen and leather armor simulants [33] Aldrete and colleagues [27] noticed rapidly diminished efficacy of bilobate bronze and iron arrows penetrating composite linen over multiple shots without resharpening.

In forensic investigations of knives used in criminal attacks, the forces recorded to penetrate cadaveric skin range from 35–55 N, followed by 35 N through subcutaneous flesh [77]. In addition to sharpness, greater velocity also reduces penetrating force due to the strain-rate sensitivity of biological tissues and armor materials [43, 51, 52, 72–74], suggesting that the forces experienced by arrows with sharp broadheads through soft tissues should be even lower than for knives. In contrast, the forces in this study for sharp broadheads to cut through two layers of thick leather are far greater (590–737 N) than knife thrusting forces in skin, demonstrating the potential efficacy of leather as an armor material. Significantly, however, the mean force of drag on the trailing shaft after broadheads in the two-blade configuration cut through just one layer of leather (48 N; Table 2) is substantially higher than for sharp knives to cut through subcutaneous flesh (35 N). This demonstrates that even after leather armor was initially defeated and the arrow continued penetrating into underlying flesh, drag from friction on the trailing shaft against the armor could remain the greatest inhibitor of further penetration. Sharp trilobate and quadrilobate arrow tips would therefore continue to outperform bilobate tips even when leather armor was directly underlain by the body of the wearer. Given the greater resistance to cutting through two layers of leather for broadheads in the four-blade configuration in this experiment, but only a marginal decrease in drag on the trailing shaft relative to three blades (Fig 8), trilobate arrow points may indeed have proven the best option for attacking lightly armored opponents. This may explain the extreme rarity of quadrilobate bronze and iron points at Neo-Assyrian [3] and Roman [7, 16] sites.

The military success of the Neo-Assyrian empire may have been due in part to the willingness of its military to experiment with new types of arms and armor [78]. During the first millennium BCE, mounted horseback riding and cavalry began to replace chariots as the primary form of transport on the battlefield–and historical records demonstrate that Neo-Assyrians were among the first outside of Inner Asia to incorporate horseback riding into their military strategies [30]. Through various relations and interactions, socketed bronze trilobate and bilobate arrow points may have entered Near Eastern arrow arsenals by the 7^th^ century BCE [3], and certainly became well-integrated into Mesopotamian arsenals by the 6^th^ century [12]. The degree to which a design change is perceived as a functional innovation is context-dependent–its adoption may depend on, among other things, manufacturing costs and the availability of proper raw materials to reproduce it [79, 80]. Nevertheless, despite being more challenging to forge than bilobate forms, tanged iron trilobate points became common in the Near East and on the Eurasian Steppe [1, 15] and were later integrated into the highly trained and innovative Roman military [2, 9, 10, 16], suggesting that their functional performance outweighed manufacturing costs across broad space.

Through time, archers and their enemies chose from a range of arrow point designs dependent on the specific targets they engaged [3, 12, 14, 15, 34]. Notably, both bilobate and trilobate points, among others, continued to be used in Eurasia into the late Medieval period and two blades may have outperformed three blades for some applications, such as defeating certain types of armor. Bodkins are known to have excelled at defeating plate metal or chainmail armor, whereas wide broadheads were ideal for hunting or attacking horses [14, 34, 53, 81]. Jones [33] found that a sharp bilobate point outperformed bodkins at penetrating dense linen and leather, although he did not test trilobate points. Riesch [14] recorded better performance of narrow bilobate points and bodkins relative to wide bilobate or trilobate points at penetrating a composite leather and wood shield, and reasoned that Merovingian archers may have chosen trilobate points for engaging softer targets. Zanier [9] also suspects that earlier iron trilobate points used by Roman archers would have been highly effective against largely unarmored Germanic warriors. Soft targets, presented by lightly armored enemies, are also suggested for iron trilobate points on the eastern steppes [15]. That bilobate and trilobate points were used together in the Near East is best indicated by a bronze mold in the British Museum that could simultaneously cast three arrow points–one bilobate and two trilobates [3, 8]. Such points are also found together with quadrilateral and triangular bronze bodkins in the Near East that may have been reserved for attacking more heavily armored opponents [3, 7, 12]. Last, socketed points are also known historically as stronger than tanged connections on impacting hard objects and can also easily release from the shaft and remain in the wound [34], which may have influenced the early adoption of socketed bronze points in the Near East, some of which had one or more defined barbs [3, 8].

Our experiment shows empirically that trilobate points could excel over bilobate points for targeting lightly armored opponents, However, we do not propose to have captured the variability in the original applications of bows and arrows, which is not the strength of a controlled experiment [57, 61]. Further tests should expand on these results by considering a greater variety of arrow point designs, targets, and various ballistic impacts that likely occurred on ancient battlefields, where arrows of different design and mass were shot from powerful military bows and impacted targets of different compositions at variably distances [e.g., 5, 14, 21, 55, 56, 82].

## 5. Conclusion

Our experiments shooting two, three, and four-bladed broadheads into clay and a thick and sturdy tooling leather demonstrates a marked difference in these target media. Clay shows reduced penetration for armatures with larger surface areas due to increased friction, such as from the attachment of multiple blades. The force of cutting resistance through two layers of leather also tends to be greater for multiple blades, however, this effect in leather is offset by the dramatic reduction in drag on the trailing shaft of the arrow for broadheads with three and four blades, resulting in significantly deeper penetration relative to two blades. This result can help explain why trilobate arrow points, first invented on the Eurasian steppe as early as the 9^th^ century BCE, spread quickly into the Near East and became increasingly popular in military contexts during the Iron Age. On battlefields where a significant number of combatants wore leather armor, such as occurred across many areas of Eurasia following the adoption of cavalry warfare, trilobate and quadrilobate points would have resulted in deeper penetration and larger wounds relative to bilobate forms. Our results suggest that cast bronze and forged iron trilobate arrow points were therefore an important adaptation to the changing realities of Eurasian warfare during the first millennium BCE and indicate that future experimental work must carefully consider a wider range of contextual factors, such as armor and mode of use, that may have influenced ancient technological choices.

## Supporting information

S1 Appendix The shot database used for the analysis. (CSV)
